# The heat is on: the impact of excessive temperature increments on complications of laser treatment for ureteral and renal stones

**DOI:** 10.1007/s00345-023-04652-0

**Published:** 2023-11-27

**Authors:** Senol Tonyali, Maximilian Ferry von Bargen, Arif Ozkan, Christian Gratzke, Arkadiusz Miernik

**Affiliations:** 1 Department of Urology, Faculty of Medicine, University of Freiburg Medical Center, Hugstetter Str. 55, 79106 Freiburg, Germany; 2 Department of Urology, Koc University Hospital, Istanbul, Turkey

**Keywords:** Urolithiasis, Endourology, Thermal Injury, Laser Lithotripsy

## Abstract

**Objective:**

Technological advancements in the field of urology have led to a paradigm shift in the management of urolithiasis towards minimally invasive endourological interventions, namely ureteroscopy and percutaneous nephrolithotomy. However, concerns regarding the potential for thermal injury during laser lithotripsy have arisen, as studies have indicated that the threshold for cellular thermal injury (43 °C) can be exceeded, even with conventional low-power laser settings. This review aims to identify the factors that contribute to temperature increments during laser treatment using current laser systems and evaluate their impact on patient outcomes.

**Materials and methods:**

To select studies for inclusion, a search was performed on online databases including PubMed and Google Scholar. Keywords such as 'temperature' or 'heat' were combined with 'lithotripsy', 'nephrolithotomy', 'ureteroscopy', or 'retrograde intrarenal surgery', both individually and in various combinations.

**Results:**

Various strategies have been proposed to mitigate temperature rise, such as reducing laser energy or frequency, shortening the duration of laser activation, increasing the irrigation fluid flow rate, and using room temperature or chilled water for irrigation. It is important to note that higher irrigation fluid flow rates should be approached cautiously due to potential increases in intrarenal pressure and associated infectious complications. The utilization of a ureteral access sheath (UAS) may offer benefits by facilitating irrigation fluid outflow, thereby reducing intrapelvic pressure and intrarenal fluid temperature.

**Conclusion:**

Achieving a balance between laser power, duration of laser activation, and irrigation fluid rate and temperature appears to be crucial for urologists to minimize excessive temperature rise.

## Introduction

Urolithiasis is a commonly encountered urological disorder, with a prevalence estimated to be between 1 and 15%. The occurrence of urolithiasis varies globally and has shown a steady rise over the past decades [1]. Medical management has proven to be insufficient for most cases of urinary stones, with surgical intervention being the primary method of treatment for symptomatic patients. While open lithotomy was once the preferred surgical approach, technological advancements have shifted the standard of care to minimally invasive endourological interventions, such as ureteroscopy and percutaneous nephrolithotomy. Shock wave lithotripsy is also considered a viable option for treating upper urinary tract stones [2].

The use of laser technology in urology dates back to Pearsons et al.'s initial experiment, where they demonstrated the effects of laser on a dog's bladder. Mulvany et al. subsequently conducted research on urinary stone fragmentation using a ruby crystal laser. However, the ruby laser proved ineffective in urology, as it produced excessive heat and vaporized stones. Over time, pulsed-wave lasers, such as Nd: YAG (FREDDY) and holmium:yttrium–aluminum-garnet (Ho: YAG), were introduced and have since become very important armamentarium in urology [3].

The threshold for cellular thermal injury is known to be 43° C and has been observed to be exceeded, even with conventional low-power laser settings, in models of renal calices and ureters. Factors such as the duration of laser activation, the quantity of irrigation fluid used, and the laser pulse energy and frequency can all contribute to cumulative heat production and, consequently, potential tissue injury during lithotripsy [4].

Through this review, we seek to identify the factors that contribute to temperature increments during laser treatment using current laser systems and evaluate their impact on patient outcomes. By doing so, we hope to develop strategies to mitigate these situations and minimize the risk of complications associated with laser treatment for urolithiasis.

## Materials and methods

For our study selection, we conducted online searches through PubMed and Google Scholar with no time restrictions, using keywords such as 'temperature' or 'heat' in combination with 'lithotripsy', 'nephrolithotomy', 'ureteroscopy', or 'retrograde intrarenal surgery', both alone and in combination. We also scanned the references in all included studies to identify any additional sources of information that could be relevant to our investigation. To ensure the consistency of our findings, we restricted the language of the eligible studies to English. All current studies related to temperature rise during lithotripsy with consistent results and studies related to prevent temperature rise were included the study. Reviews of the related topics were excluded from the study. Author and year of the studies, part of urinary system assessed for temperature rise, models for the experiments, type of laser, laser fiber size, laser settings, duration of activation of laser, irrigation fluid flow rate, and temperature status were recorded.

## Literature review

### Type of laser and temperature

In another study, Molina et al. [2] investigated the temperature profiles of two laser systems, the 120 W Ho: YAG (model P120, Lumenis Ltd., Israel) and the 60 W SPTF (SOLTIVETM, Olympus, MA), during ureteral lithotripsy using an ex vivo porcine kidney and ureter model. The irrigation was conducted with saline at room temperature (27 °C) using a manual pumping system. The authors reported that both laser systems' temperature profiles remained below the threshold for potential cellular injury (43 °C) during the experiments. Moreover, at dusting settings (0.3 J, 70 Hz, Long Pulse), the Ho: YAG laser's median temperature increase was higher than that of the SPTF laser (40.6 °C vs. 35.8 °C, respectively, *p* = 0.064).

Belle et al. [6] investigated the temperature differences between thulium fiber laser (TFL) and Ho: YAG lasers during ureteral stone lithotripsy. TFL was found to produce the highest temperature at all laser settings of 3.6 W, 10 W, and 30 W compared to the 30 W Medilas H Solvo HL (Dornier, Munich, Germany) and 100 W Empower HL (Olympus, MA) lasers. There was no significant difference between the TFL (40.93 °C) and the Empower HL (41.17 °C, *p* > 0.05) at 20 W. At 30 W, the TFL produced a significantly high maximum temperature of 44.37 °C and exceeded the threshold.

Hardy et al. [10] compared the effects of Ho: YAG and TFL on temperatures in an in vitro ureteral model. They used Ho: YAG laser at 600 mJ, 350 μs, 6 Hz, and TFL at 35 mJ, 500 μs, 150–500 Hz settings. Saline irrigation flow rates were 22.7 ml/min for TFL and 13.7 ml/min for Holmium laser. They found a mean peak irrigation fluid temperature of 24 ± 1 °C for Holmium and 33 ± 3 °C, 33 ± 7 °C, and 39 ± 6 °C for TFL at pulse rates of 150, 300, and 500 Hz, respectively. The researchers concluded that to avoid excessive thermal rise and provide a sufficient safety margin, TFL lithotripsy should be performed with pulse rates under 500 Hz and/or with increased irrigation rates.

In another experimental study, Sierra et al. [15] compared the safety of Ho: YAG and TFL in an in vivo porcine model and in vitro ureteral model. The temperature reached 40.2 °C with TFL and 41 °C with Ho: YAG laser at max 20W power with gradually decreased irrigation (40 cmH_2_O continuous).

Similarly, Taratkin et al. [16] investigated the temperature profile between Ho: YAG laser and a novel TFL during laser lithotripsy in an in vitro model. The researchers reported that both lasers yielded similar temperature increases, and the amount of energy used was equivalent to the amount of heat introduced into the system. Temperature increase at 20W without irrigation was 10.9 ± 0.5 °C for Ho: YAG and 11.0 ± 0.5 °C for super-pulsed TFL.

### Laser settings, irrigation flow rate and temperature

In their study, Wollin et al. [1] examined the impact of laser settings and irrigation flow rates on ureteral temperature during ureterolithotripsy in an in vitro setting. Specifically, they utilized a 200 μm laser with varying pulse energies and frequencies (0.2 J/50 Hz, 0.6 J/6 Hz, 0.8 J/8 Hz, 1 J/10 Hz, and 1 J/20 Hz) for a duration of 60 s within a tubing system that allowed for specified room temperature flow rates (100, 50, and 0 mL/minute). Their findings indicated that at an irrigation rate of 100 mL/min, only the highest laser setting (1 J/20 Hz) produced a maximum temperature of 30.7C, which was deemed clinically insignificant. However, with no irrigation fluid flow, all maximum temperatures exceeded the threshold for cellular thermal injury (43 °C). Furthermore, at the highest laser setting (1 J/20 Hz) with no irrigation, the maximum temperature exceeded 100 °C in their study [1].

Molina et al. [3] investigated the temperature profile of Ho: YAG laser lithotripsy in the urinary tract of Ovis Aries (sheep) with and without irrigation. They reported that the temperature rise was significantly higher in the intact ureteral model without irrigation compared to with irrigation, with temperatures of 49.5 ± 2.3 °C versus 37.4 ± 2.5 °C, respectively.

Aldoukhi et al. [5] investigated the caliceal fluid temperature during high-power Ho: YAG lithotripsy in an in vitro porcine model. They used the Ho: YAG laser at a 0.5 J × 80 Hz = 40 W setting with high, medium, or no irrigation. The peak temperatures for no, medium, and high irrigation were 84.8 °C, 63.9 °C, and 43.6 °C, respectively.

Hein et al. [11] utilized an in vitro model to simulate the renal pelvis and assess the changes in temperature during Ho: YAG lithotripsy. The results indicated that without irrigation, laser application led to a rapid temperature rise of up to Δ28 K, increasing to 68 °C at 100 W. However, higher irrigation rates resulted in a lower temperature increase. At the highest irrigation rates of 100 ml/min and the highest laser power setting (100 W), the temperature increased by only 5 K.

Teng et al. [17] conducted a study to assess the changes in irrigation fluid temperature in renal calyces during Ho: YAG lithotripsy using flexible URS in a real-life scenario. The authors found that at a power setting of 1 J/20 Hz and an irrigation flow rate of 15 ml/min, the temperature rise was significantly higher than other groups. Moreover, the temperature increase was significantly higher in groups with lower irrigation flow rates at the same laser settings. The time required to reach a temperature of 43 °C at 1 J/20 Hz was significantly shorter than that at 0.5 J/20 Hz when there was no irrigation.

In another study, Winship et al. [19] investigated the temperature rise in a benchtop ureteral model using both flexible and semi-rigid ureteroscopes with Ho: YAG laser. Laser energy was delivered at various settings, and with irrigation at 100 mm Hg using the semi-rigid scope, 1 J/20 Hz was the only laser setting to produce a temperature rise over 6 °C. The temperature returned to close to baseline levels within 2 s after laser cessation.

Aldoukhi et al. [4] investigated the effect of pedal activation on fluid temperature and thermal profiles in an in vitro experimental caliceal model using the Ho: YAG laser (pulse120; Lumenis). They reported that longer pedal activation times led to higher peak temperatures and thermal effects. The thermal injury threshold (43 °C) was reached in 9 s when 40 W was applied at 50% operator duty cycle (ODC = lasing time/lithotripsy time) with laser activation patterns of 30 s on/off and 15 s on/off.

Wriedt et al. [20] evaluated tissue heating with magnetic resonance imaging in an ex vivo porcine kidney model using different lithotripsy parameters. The authors reported that an irrigation rate of at least 70 ml/min is necessary to avoid exceeding 120 min CEM_43_ when using a laser application of 30 W for 10 s. Additionally, they observed a focal temperature rise on the calyx wall in experiments with human stones, which they attributed to non-elimination of heated fragmented stone pieces from the surgical area in the kidney.

### Pelvicalyceal volume and temperature

Khajeh et al. [13] studied the relationship between pelvicalyceal volume and temperature rise during Ho: YAG laser lithotripsy. The findings showed that the temperature elevation and thermal dose from laser activation were inversely related to the fluid volume in each model and the irrigation rate. The safe temperatures below the threshold of tissue injury were obtained at an irrigation rate of 40 mL/min during 1 min of continuous laser activation in all models [13].

### Types of pulse modulation and temperature

In a study by Peteinaris et al. [14], the effects of the newly introduced Holmium pulse modulation system MOSES were compared with conventional pulse delivery technology on irrigation fluid temperature (IFT) during flexible ureteroscopy in a live-anesthetized porcine model. The study considered the threshold for a dangerous IFT as 54 °C. The results revealed that both the MOSES and conventional laser activation at 60 W led to a significant temperature rise, exceeding the safety threshold of 54 °C in approximately 10 s and reaching a hazardous temperature of 66.4 °C in 18 s under gravity irrigation. However, the IFT did not exceed 54 °C at any settings with manual pump irrigation.

Winship et al. [18] investigated the effect of pulse type of Ho: YAG (short pulse (SP), long pulse (LP), MOSES contact (Mc), MOSES distance (MD)) on irrigation fluid in an experimental ureteral model. The authors reported that LP produced the greatest temperature change from baseline at a 1 J/20 Hz setting, and thermal dose exceeded the injury threshold for all pulse types in < 3 s of activation at the same setting.

### Formula to suggest temperature rise

In a separate study, Hein et al. [12] examined the thermal effects of Ho: YAG laser during retrograde intrarenal surgery and percutaneous nephrolithotomy in an ex vivo porcine kidney model. The researchers suggested a formula to calculate temperature changes with irrigation rates higher than 30 ml/min: ΔT = 15 K × (power [W]/ irrigation [ml/min]) based on their results.

Lastly, Williams et al. [21] developed a mathematical model to predict laser-induced temperature alterations in a kidney during lithotripsy. The model was based on renal volume, irrigation flow rate, irrigation fluid temperature, and laser power. The researchers cautioned clinicians to use room temperature irrigation fluids for irrigation and avoid extensive laser usage. They also suggested altering the irrigation fluid rate by adjusting the inflow (i.e., pressured irrigation fluid) or outflow (i.e., usage of UAS).

### Possible preventive measures for temperature rise

In their study, Dau et al. [7] investigated the effect of chilled irrigation on caliceal fluid temperature and time to reach the thermal injury threshold in an in vitro model. The experiments utilized a 120 W Ho:YAG laser (pulse120; Lumenis, CA) at a 0.5 J · 80 Hz (40 W) setting in short pulse mode for 60 s. The thermal injury threshold was reached in 28 s with room temperature (19 °C) irrigation and in 33 s with chilled irrigation (1 °C) with a flow rate of 8 mL/min. With 12 mL/min irrigation, the threshold was reached in 46 s with room temperature irrigation, but it was not reached with chilled irrigation. In another study, they evaluated and compared the thermal effects of chilled irrigation (4 °C–1 °C), room temperature (RT) (20 °C–1 °C), and warm (WM) irrigation (37 °C–1 °C) during ureteroscopy with laser activation in an in vivo porcine model. The researchers concluded that irrigation with chilled saline during ureteroscopic laser lithotripsy decelerates temperature rise, decreases peak temperature, and lengthens the time to thermal injury compared with irrigation with room temperature or warm saline solutions [7].

In their study, Gallegos et al. [8] reported that using a ureteral access sheath (UAS) provides lower intrarenal temperatures regardless of laser configuration and irrigation solution height. The rise in temperature obtained per minute with an irrigation height of 50 cm H_2_O, an energy of 1 J, and a frequency of 15 Hz was higher when UAS was not utilized (5.8 °C versus 3.8 °C) [8]. Similarly, Noureldin et al. [9] investigated the effects of irrigation rates and UAS size on intrarenal temperature during flexible ureteroscopy in a live-anesthetized porcine model. They found that fURS under gravity irrigation (the fluid bag at 1 m over the operation table) without UAS was associated with hazardous intrarenal temperatures (54 °C) even at a laser power as low as 20 W for as short as 20 s of laser activation. With pump irrigation, if the laser was activated at the highest power setting (60 W) for 60 s, the intrarenal temperature remained on the safe side, even without the use of UAS [9] (Table 1).

**Table 1:** Descriptives and significant results of studies on effect of lasers on intrarenal temperatures

Author and year	Assessed area	Model for the experiments	Laser type	Fiber size	Laser settings	Duration of laser activation	Irrigation fluid flow rate	Maximum temperature
Molina WR, 2021	Ureter	Ex vivo porcine ureter-kidney	Ho:YAG laser (model P120, Lumenis Ltd., Israel)	200 μm	Dusting: 0.3 J, 70 Hz, Long Pulse. Fragmentation: 0.8 J, 8 Hz, Short Pulse	5 s	Manual pumping system	Median temperature at fragmentation: 30.0 °C for Ho: YAG, 33.3 °C for SPTFL, * p* = 0.004 Median temperature dusting: 40.6 °C for Ho: YAG vs 35.8 °C for SPTFL, * p* = 0.064
			Super-pulsed Thulium Fiber (SPTFL) (SOLTIVE ™_,_Olympus, MA)		Dusting: 0.1 J, 200 Hz, SP Fragmentation: 0.8 J, 8 Hz, Short Pulse (SP) and			
Molina WR, 2015	Ureter	Ex vivo sheep ureter model	Ho:YAG laser (Dornier Medilas, Kennesaw, GA)	365 μm	10W = 1 J × 10 Hz	3 s	8 mL/s	Temperatures obtained: 37.4 °C with irrigation and 49.5 °C without irrigation The thermal spread along the external ureter wall: 0.9 ± 0.1 cm with irrigation and 1.1 ± 0.1 cm without irrigation
Aldoukhi AH, 2021	Kidney	Human and in vitro calyceal model	Ho: YAG (pulse 120; Lumenis, CA)	242 μm	1200 J for 60 s in five different patterns: 0.5 J·80 Hz (40 W) at 50% ODC (30 s on/30 s off), 0.5 J · 80 Hz (40 W) at 50% ODC (15 s on/15 s off · 2), 0.5 J · 80 Hz (40 W) at 50% ODC (10 s on/10 s off · 3), 0.5 J · 80 Hz (40 W) at 50% ODC (5 s on/5 s off · 6), 0.5 J · 40 Hz (20 W) at 100% ODC	ODC(Operator duty cycle) = lasing time/lithotripsy time	Peristaltic pump (Masterflex; Cole Parmer, IL) at 8 mL/min	t_43_ = 120 equivalent minutes, was reached after 9 s when the laser was activated at 40 W with 30 s on/off and 15 s on/off patterns
Aldoukhi AH, 2018	Kidney	In vivo porcine	Ho: YAG (pulse 120; Lumenis, CA)	242 μm	40 W (0.5 J, 80 Hz, short pulse)	100 s	High irrigation: pressure bag inflated to 150 mmHg and placed at 100 cm height, equivalent to 304 cm of gravity irrigation Medium irrigation consisted of a saline bag at 100 cm height No irrigation	t_43_ was exceeded for all trials with no irrigation. The mean time to reach the threshold of thermal injury: - 12.7 s (range 2–37 s) for no irrigation - 17.8 s (range 5–30 s) for medium irrigation
Belle JD, 2022	Ureter	3-dimensional printed kidney-ureter model	Dornier 30 W Ho:YAG	200 μm	3.6, 10, 20, and 30 W	60 s	35 cc/min	
			Olympus 100 W Ho: YAG					At 30 W, the TFL exceeded 43 °C
			Olympus 60 W TFL					The TFL generated higher average ureteral fluid temperatures than the Dornier and Empower HL at all power settings tested
Dau JJ, 2021	Kidney	In vitro caliceal mode	Ho: YAG (pulse 120; Lumenis, CA)	242 μm	0.5 J · 80 Hz (40 W)	60 s	0, 8, 12, 15, and 40 mL/minute	For trials using RT irrigation, average peak temperatures were: 58.6 °C ± 0.4 °C for 8 mL/min, 53.1 °C ± 0.4 °C for 12 mL/min, 48.5 °C ± 0.2 °C for 15 mL/min, 32.9 °C ± 0.2 °C for 40 mL/min
								For trials using CH irrigation were: 56.4 °C ± 0.4 °C for 8 mL/min, 48.0 °C ± 0.2 °C for 12 mL/min, 43.0 °C ± 0.5 °C for 15 mL/min, 20.7 °C ± 0.2 °C for 40 mL/min
Dau JJ, 2022	Kidney	Anesthetized pigs	Ho: YAG (pulse 120; Lumenis, CA)	230 μm (Moses 200 D/F/L; Lumenis)	30 W (0.5 J·60 Hz, short pulse)	60 s	8, 12 and 15 mL/minute	Mean calculated thermal dose was inversely related to irrigation rate and was generally highest with WM irrigation and lowest with CH irrigation The mean time to threshold of thermal injury was 9, 15, and 23 s for WM, RT, and CH irrigation, respectively, at 8 mL/min irrigation
Gallegos H, 2021	Kidney	ex vivo porcine model	Ho: YAG	275 μm	Frequencies: 5, 10, and 15 Hz X Energies: 0.5, 0.8, 1, and 1.5 J	60 s	8.5, 9.5, and 11.1 mL/min	Irrigation height was founded to be significant in the DT determination. 50 cm H_2_O irrigation height decreased the DT by 4.8 °C compared to 30 cm H_2_O independent of the use of access sheath and laser configuration The use of an access sheath provides lower intrarenal temperatures regardless of laser configuration and water height
Noureldin YA, 2020	Kidney	Live-anesthetized porcine model	Ho: YAG (pulse 120; Lumenis, CA)	200 μm	20 W, 40 W, and 60 W	60 s	1 m gravity irrigation or manual pump irrigation 1 pump every 3 s	When UAS 12/14 was used the maximum IRT was detected equal to 63.4 °C at 40 W and equal to 74.5 °C at 60 W with gravity irrigation
								With manual pump irrigation temperatures reaching the threshold of 54 °C were not noted regardless of the laser power setting with or without the presence of a UAS
Hardy LA, 2015	Ureter	In vitro ureter model	100 W TFL (IPG Photonics, Oxford, MA)	100–244 μm	Pulse energy of 35 mJ, pulse duration of 500 μs, and pulse rates of 150, 300, or 500 Hz	As much as required for complete stone ablation	22.7 ml/min	33 ± 3 °C, 33 ± 7 °C, and 39 ± 6 °C for TFL at pulse rates of 150, 300, and 500 Hz, respectively
			20 W Ho: YAG laser (Medilas H20, Dornier MedTech, Wessling, Germany)	270–464 μm	600 mJ, 350 μs, and 6 Hz	As much as required for complete stone ablation	13.5 ml/min	Mean peak saline temperature: 24 ± 1 °C
Hein S, 2018	Kidney	In vitro kidney model	100 W Ho: YAG	272 μm or 940 μm	-15 W (0.5 J/30 Hz/150 μs) and 45 W (1.5 J/30 Hz/150 μs) -18 W (1.0 J/18 Hz/400 μs vs. 4.5 J/4 Hz/400 μs) and 54 W (2.0 J/2 Hz/400 μs vs. 4.5 J/12 Hz/400 μs) -18 W (4.5 J/4 Hz/400 μs) and 100 W (4.5 J/22.3 Hz/400 μs)	120 s	0, 5, 10, 16.65, 30, 40, 50, 75, and 100 ml/min	Laser application without irrigation results in a quick increase in temperature of up to ∆28 K, rising to 68 °C at 100W High irrigation rates of 100 ml/min result in a temperature rise of 5 K at 100 W -During experiments with renal calculi, the temperature rise was quicker, more turbulent, and 12.9% higher than that reached in the blank test
Hein S, 2020	Kidney	Ex vivo porcine kidney model	100-W Ho: YAG laser (Sphinx, LISA laser products OHG, Katlenburg-Lindau, Germany)	272 μm, 550 μm, 940 μm	Varying between 5 and 100 W	Na	0–100 ml/min	ΔT for irrigation rates ≥ 30 ml/min: ΔT = 15 K × (power [W]/irrigation [ml/min])
Khajeh NR, 2022	Kidney	In vitro experimental model	Ho: YAG	230 μm	40 W (0.5 J · 80 Hz, SP)	60 s	0, 8, 15, and 40 mL/min	Peak temperature and rise time were inversely related to irrigation rate -Smaller volumes receive greater thermal dose and reach threshold of tissue injury more rapidly than larger volumes
Peteinaris A, 2022	Kidney	Live-anesthetized porcine model	Ho: YAG (pulse 120; Lumenis, CA)	200 μm	20W (1 J, 20 Hz), 40W (1 J, 40 Hz), 60 W (2 J, 30 Hz) MOSES™ pulse delivery vs conventional pulse delivery technology	30 s	1 m Gravity irrigation	For 60 W both the MOSES and the conventional laser exceeded the safety threshold of 54 °C in approximately 10 s reaching the hazardous temperature of 66.4 °C in 18 s
							Manual irrigation set at 1 pump every 3 s	For 60 W, the MOSES laser was close to a highest temperature of 42 °C and the standard laser close to 44 °C throughout the 30 s recording
Sierra A, 2023	Kidney and Ureter	In vivo porcine model	Ho: YAG (Cyber: Ho 150 WTM)	200 μm	0.5 J/10 Hz, 0.5 J/20 Hz, 0.7 J/10 Hz, 0.7 J/20 Hz, 1 J/12 Hz, 1 J/20 Hz	3 min, 7 min, 10 min	Continuous irrigation at 40 cmH_2_O	TFL is safe when working with 20 W in the kidney and 12 W in the ureter
			TFL (Fiber dust)					
Taratkin M, 2020	Not specified	In vitro experimental model	Ho:YAG (100 W, Lumenis, Yokneam, Israel	200 μm	0.2 J X 40 Hz (8 W)	60 s	Precise flow control 0, 10, 35 mL/min	With no flow temperature rise: 10.9 ± 0.5 At 35 mL/min ^∆T^local: 2.8 ± 0.5
			Super-pulse NTO IRE-Polus, Russia					With no flow temperature rise: 11 ± 0.5 At 35 mL/min ^∆T^local: 1.9 ± 0.5
Teng JF, 2021	Kidney	Human beings	Ho: YAG	200 μm	0.5 J/20 Hz and 1 J/20 Hz	60 s	0, 15 ml/min or 30 ml/ min	Temperature rise: 1 J/20 Hz, 15 ml/min: 4.91 ± 0.58 0.5 J/20 Hz, 30 ml/min:1.50 ± 0.44,
Winship B, 2019	Ureter	Benchtop ureteral model	Ho: YAG (Pulse 120H; Lumenis, Yokneam, Israel	365 μm	0.6 J/6 Hz, 0.8 J/8 Hz, 1 J/10 Hz, 1 J/20 Hz, 0.2 J/80 Hz	45 s	0-, 100-, and 200-mm Hg	Flexible scope: At 200 mm Hg, changes > 6 °C occurred at 1 J/10 Hz (15 s), 0.2 J/80 Hz (3 s), and 1 J/20 Hz (2 s) At 0 mm Hg, all settings produced changes > 6 °C within 3 s, except 0.6 J/6 Hz (35 s)
Wriedt R, 2023	Kidney	Ex vivo porcine kidney model	Ho: YAG (Sphinx Junior 30 W, LISA, Germany)	550 μm	14 W = 1.2 J, 12 Hz, 90 μs pulse duration 30 W = 3.3 J, 9 Hz, 230 μs pulse duration	5 or 10 s	10, 30, 50, 70 and 100	At high irrigation rates (I) of I ≥ 50 ml/min, CEM_43_ remained below the threshold of 120 min at any setting except for 10 s laser/10 s delay
Williams JG, 2021	Kidney	Experimental model	Ho: YAG					Mathematical model

WM irrigation: Warmed irrigation, RT irrigation: Room temperature irrigation, CH irrigation: Chilled İrrigation, DT: Delta temperature, ^∆T:^ Temperature change

## Discussion

Numerous studies have been conducted on temperature changes during laser lithotripsy using various experimental models and lasers. There are several factors that may contribute to temperature rise, including the type of laser, laser settings (high power, high frequency), irrigation fluid rate, laser activation time, amount of fluid in the space, and stone particles collecting heat during lithotripsy. The two main lasers currently used in endoscopic lithotripsy are Ho: YAG and Thulium lasers, and each laser system has undergone several modifications over the years. The power of these lasers has increased significantly, and the most recent innovations in lasers for lithotripsy include pulse modulation using MOSES technology for Ho: YAG and pulsed Thulium YAG lasers, as well as Thulium fiber lasers.

Increased power leads to higher temperature rise, and clinicians must balance multiple variables to avoid adverse effects. To mitigate temperature rise, modifications such as decreasing the laser energy or frequency, decreasing the duration of laser activation, increasing the irrigation fluid flow rate, and using room temperature or chilled water for irrigation may be considered.

While higher irrigation fluid flow rates may be an option to decrease temperature rise, the increased intrarenal pressure and related infectious complications must be taken into consideration. Pyelo-venous reflux may occur when the intrapelvic renal pressure exceeds 30–35 mmHg [22], so when increasing the flow rate or heightening the irrigation fluid bag, this phenomenon must be considered. Ureteral access sheath (UAS) use may facilitate irrigation fluid outflow and decrease not only intrapelvic pressure but also intrarenal fluid temperature [9], but UAS use may also cause complications like ureteral damage and stricture. Using novel pressure-controlled suctioning UAS may overcome this issue [23], but further studies are warranted to determine its utility.

Laser settings and lasing time are the primary determinants of heat production, and the total energy created is calculated based on the formula "Total energy = pulse energy x frequency." Thus, special care must be given to the lasing time, particularly under low irrigation rates, when working with high pulse energy and/or frequency.

The fluid volume at the lased area is also related to increased temperature. With the same applied energy, less fluid will be heated more quickly. Therefore, excessive temperature rise must be kept in mind when using the laser in a relatively small space such as a renal calyx or ureter.

In addition to the aforementioned issues, the initial temperature of the irrigation fluid is another factor that can contribute to temperature rise during laser lithotripsy. It has been demonstrated that the use of room temperature irrigation fluid during endoscopic surgeries may result in a decrease in core body temperature, even leading to hypothermia. The absorption of irrigation fluid and the circulation of blood through the surgical site exposed to a large volume of room temperature irrigation fluid can cause a decrease in the patient's core body temperature. Hypothermia is a serious and sometimes troublesome reality, particularly for anesthesiologists. Therefore, according to the results of a meta-analysis, it was recommended to use warmed irrigation fluid to decrease heat loss and the risk of perioperative shivering and hypothermia [24]. However, when trying to prevent hypothermia, one must also be aware of excessive temperature rise during laser lithotripsy. This is because all newly generated heat by the laser would be added to the basal fluid temperature, which might facilitate exceeding 43 °C, the threshold for tissue damage.

Ultimately, the issue of uncontrolled temperature increase during laser lithotripsy is multifactorial and requires careful consideration of various parameters, including laser settings, irrigation fluid rate and temperature, and fluid volume at the lased area. To address this issue, future research is needed to develop laser systems with improved temperature control mechanisms and to explore novel methods for monitoring physical conditions during laser energy application. Additionally, the development of advanced monitoring systems is of utmost importance to allow physicians to monitor temperature changes and other physical conditions during endourology procedures. With these advancements, we can ensure safer and more effective procedures, minimizing the risk of tissue damage and other adverse events associated with uncontrolled temperature increase.

## Limitations

Our study is not without limitations. First of all, this is a narrative review not a systematic review aiming to take a snapshot of current literature. Given the wide extension of the topic, it is hard to conduct a systematic review, since there are too many variables effecting temperature alterations during lithotripsy which must take into consideration. It was also not possible to pool the data and perform a meta-analysis, because there was no structured setup for the experiments and study design was different in almost all studies. The used equipment i.e., device for temperature measurement was different and some studies were in vitro studies using animal kidneys and some were experimental settings mimicking kidney anatomy.

## Conclusion

With the advancement of endoscopic instruments and progress in laser technology, endoscopic surgery for stone removal has become the preferred treatment for urolithiasis. High-powered lasers enable the treatment of large stones through retrograde intrarenal surgery or percutaneous nephrolithotomy. Nevertheless, high power required for rapid disintegration also entail limitations such as increased intrarenal temperature, poor vision caused by the snow-storm effect during dusting and prolonged operation times, particularly for retrograde intrarenal surgery. To address excessive temperature rise, urologists must establish a balance between laser power, lasing time, and irrigation fluid rate and temperature.
